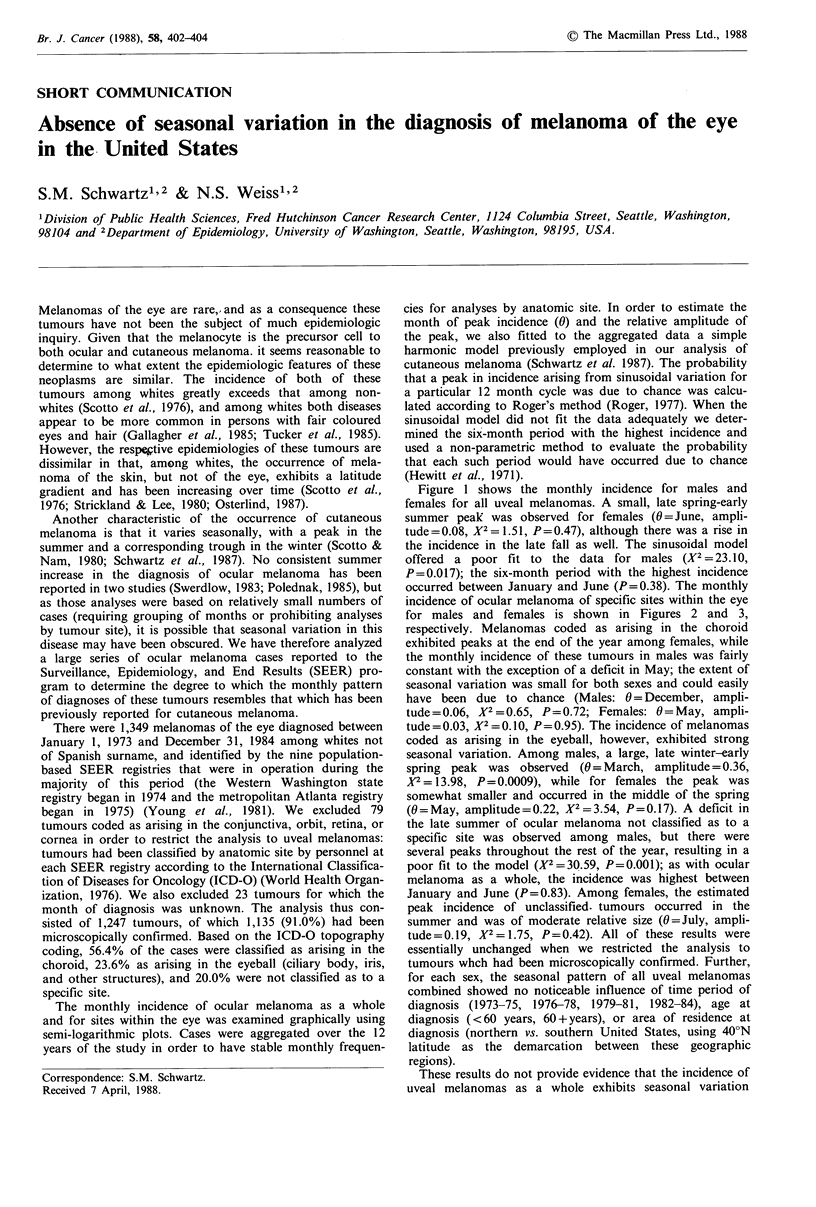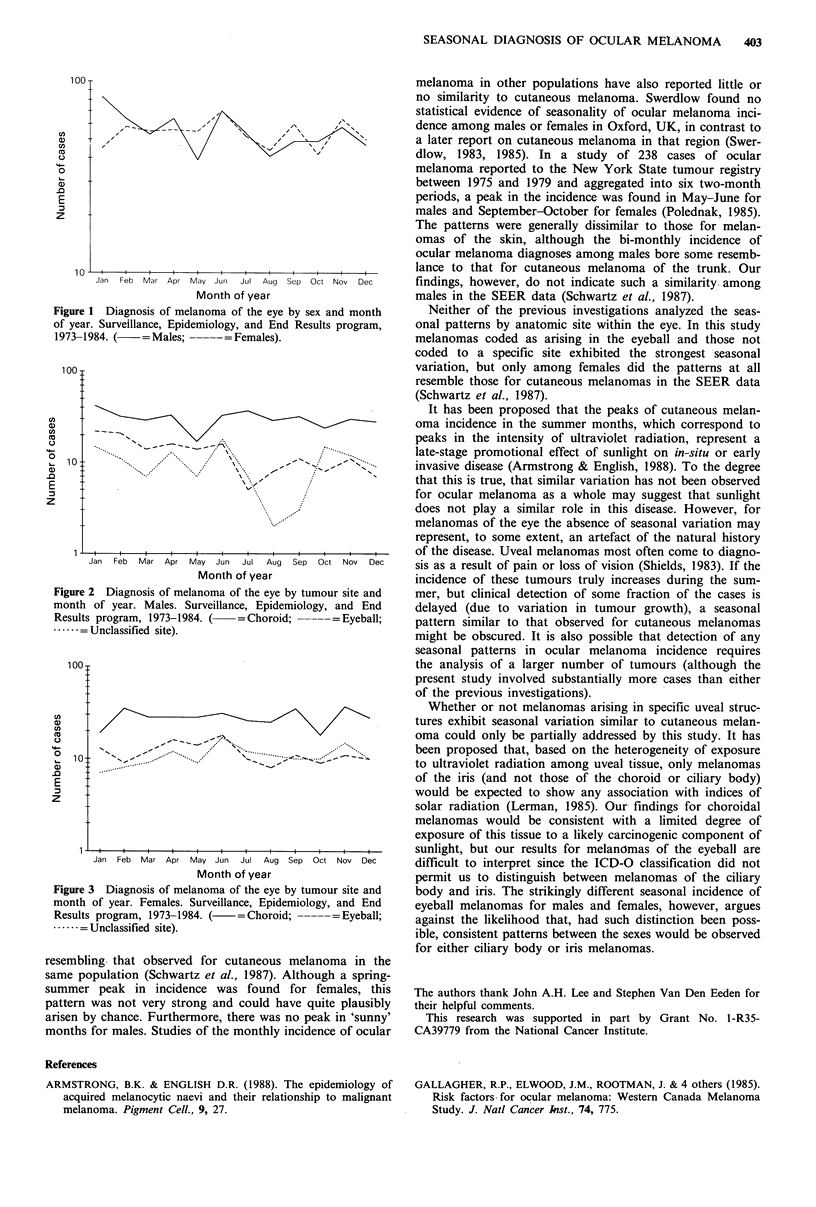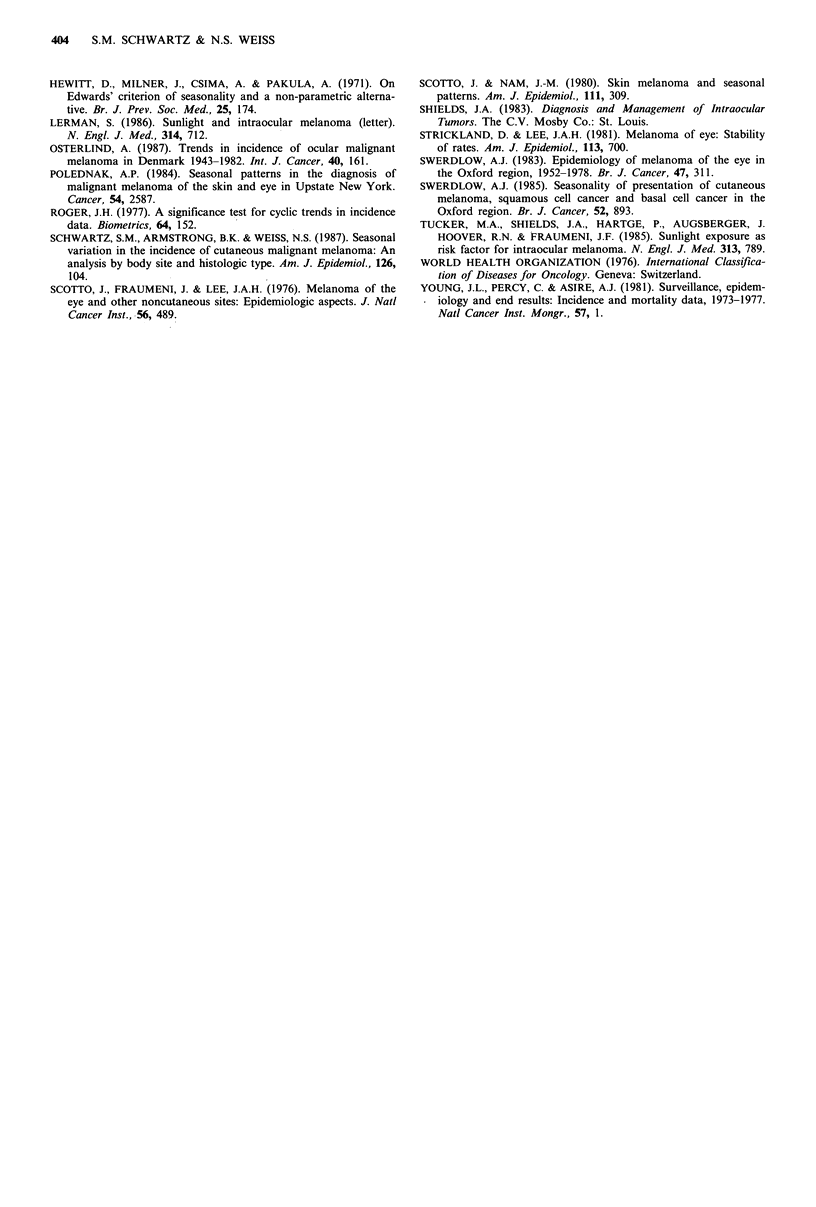# Absence of seasonal variation in the diagnosis of melanoma of the eye in the United States.

**DOI:** 10.1038/bjc.1988.228

**Published:** 1988-09

**Authors:** S. M. Schwartz, N. S. Weiss

**Affiliations:** Division of Public Health Sciences, Fred Hutchinson Cancer Research Center, Seattle, Washington 98104.


*Br. J. Cancer* (1988), **58**, 402–404 © The Macmillan Press Ltd., 1988

**SHORT COMMUNICATION**

### Absence of seasonal variation in the diagnosis of melanoma of the eye in the United States

S.M. Schwartz[1,2] & N.S. Weiss[1,2]

[1]*Division of Public Health Sciences, Fred Hutchinson Cancer Research Center, 1124 Columbia Street, Seattle, Washington, 98104 and* [2]*Department of Epidemiology, University of Washington, Seattle, Washington, 98195, USA.*

Melanomas of the eye are rare, and as a consequence these tumours have not been the subject of much epidemiologic inquiry. Given that the melanocyte is the precursor cell to both ocular and cutaneous melanoma. it seems reasonable to determine to what extent the epidemiologic features of these neoplasms are similar. The incidence of both of these tumours among whites greatly exceeds that among non-whites (Scotto *et al.*, 1976), and among whites both diseases appear to be more common in persons with fair coloured eyes and hair (Gallagher *et al.*, 1985; Tucker *et al.*, 1985). However, the respective epidemiologies of these tumours are dissimilar in that, among whites, the occurrence of melanoma of the skin, but not of the eye, exhibits a latitude gradient and has been increasing over time (Scotto *et al.*, 1976; Strickland & Lee, 1980; Osterlind, 1987).

Another characteristic of the occurrence of cutaneous melanoma is that it varies seasonally, with a peak in the summer and a corresponding trough in the winter (Scotto & Nam, 1980; Schwartz *et al.*, 1987). No consistent summer increase in the diagnosis of ocular melanoma has been reported in two studies (Swerdlow, 1983; Polednak, 1985), but as those analyses were based on relatively small numbers of cases (requiring grouping of months or prohibiting analyses by tumour site), it is possible that seasonal variation in this disease may have been obscured. We have therefore analyzed a large series of ocular melanoma cases reported to the Surveillance, Epidemiology, and End Results (SEER) program to determine the degree to which the monthly pattern of diagnoses of these tumours resembles that which has been previously reported for cutaneous melanoma.

There were 1,349 melanomas of the eye diagnosed between January 1, 1973 and December 31, 1984 among whites not of Spanish surname, and identified by the nine population-based SEER registries that were in operation during the majority of this period (the Western Washington state registry began in 1974 and the metropolitan Atlanta registry began in 1975) (Young *et al.*, 1981). We excluded 79 tumours coded as arising in the conjunctiva, orbit, retina, or cornea in order to restrict the analysis to uveal melanomas: tumours had been classified by anatomic site by personnel at each SEER registry according to the International Classification of Diseases for Oncology (ICD-O) (World Health Organization, 1976). We also excluded 23 tumours for which the month of diagnosis was unknown. The analysis thus consisted of 1,247 tumours, of which 1,135 (91.0%) had been microscopically confirmed. Based on the ICD-O topography coding, 56.4% of the cases were classified as arising in the choroid, 23.6% as arising in the eyeball (ciliary body, iris, and other structures), and 20.0% were not classified as to a specific site.

The monthly incidence of ocular melanoma as a whole and for sites within the eye was examined graphically using semi-logarithmic plots. Cases were aggregated over the 12 years of the study in order to have stable monthly frequencies for analyses by anatomic site. In order to estimate the month of peak incidence ($\theta$) and the relative amplitude of the peak, we also fitted to the aggregated data a simple harmonic model previously employed in our analysis of cutaneous melanoma (Schwartz *et al.* 1987). The probability that a peak in incidence arising from sinusoidal variation for a particular 12 month cycle was due to chance was calculated according to Roger's method (Roger, 1977). When the sinusoidal model did not fit the data adequately we determined the six-month period with the highest incidence and used a non-parametric method to evaluate the probability that each such period would have occurred due to chance (Hewitt *et al.*, 1971).

Figure 1 shows the monthly incidence for males and females for all uveal melanomas. A small, late spring-early summer peak was observed for females ($\theta$=June, amplitude=0.08, $X^2=1.51$, $P=0.47$), although there was a rise in the incidence in the late fall as well. The sinusoidal model offered a poor fit to the data for males ($X^2=23.10$, $P=0.017$); the six-month period with the highest incidence occurred between January and June ($P=0.38$). The monthly incidence of ocular melanoma of specific sites within the eye for males and females is shown in Figures 2 and 3, respectively. Melanomas coded as arising in the choroid exhibited peaks at the end of the year among females, while the monthly incidence of these tumours in males was fairly constant with the exception of a deficit in May; the extent of seasonal variation was small for both sexes and could easily have been due to chance (Males: $\theta$=December, amplitude=0.06, $X^2=0.65$, $P=0.72$; Females: $\theta$=May, amplitude=0.03, $X^2=0.10$, $P=0.95$). The incidence of melanomas coded as arising in the eyeball, however, exhibited strong seasonal variation. Among males, a large, late winter–early spring peak was observed ($\theta$=March, amplitude=0.36, $X^2=13.98$, $P=0.0009$), while for females the peak was somewhat smaller and occurred in the middle of the spring ($\theta$=May, amplitude=0.22, $X^2=3.54$, $P=0.17$). A deficit in the late summer of ocular melanoma not classified as to a specific site was observed among males, but there were several peaks throughout the rest of the year, resulting in a poor fit to the model ($X^2=30.59$, $P=0.001$); as with ocular melanoma as a whole, the incidence was highest between January and June ($P=0.83$). Among females, the estimated peak incidence of unclassified tumours occurred in the summer and was of moderate relative size ($\theta$=July, amplitude=0.19, $X^2=1.75$, $P=0.42$). All of these results were essentially unchanged when we restricted the analysis to tumours whch had been microscopically confirmed. Further, for each sex, the seasonal pattern of all uveal melanomas combined showed no noticeable influence of time period of diagnosis (1973–75, 1976–78, 1979–81, 1982–84), age at diagnosis (<60 years, 60+years), or area of residence at diagnosis (northern *vs.* southern United States, using 40°N latitude as the demarcation between these geographic regions).

These results do not provide evidence that the incidence of uveal melanomas as a whole exhibits seasonal variation

Correspondence: S.M. Schwartz.
Received 7 April, 1988.

**Figure 1** Diagnosis of melanoma of the eye by sex and month of year. Surveillance, Epidemiology, and End Results program, 1973–1984. (—— = Males; ----- = Females).

**Figure 2** Diagnosis of melanoma of the eye by tumour site and month of year. Males. Surveillance, Epidemiology, and End Results program, 1973–1984. (—— = Choroid; ----- = Eyeball; ······ = Unclassified site).

**Figure 3** Diagnosis of melanoma of the eye by tumour site and month of year. Females. Surveillance, Epidemiology, and End Results program, 1973–1984. (—— = Choroid; ----- = Eyeball; ······ = Unclassified site).

resembling that observed for cutaneous melanoma in the same population (Schwartz *et al.*, 1987). Although a spring-summer peak in incidence was found for females, this pattern was not very strong and could have quite plausibly arisen by chance. Furthermore, there was no peak in 'sunny' months for males. Studies of the monthly incidence of ocular melanoma in other populations have also reported little or no similarity to cutaneous melanoma. Swerdlow found no statistical evidence of seasonality of ocular melanoma incidence among males or females in Oxford, UK, in contrast to a later report on cutaneous melanoma in that region (Swerdlow, 1983, 1985). In a study of 238 cases of ocular melanoma reported to the New York State tumour registry between 1975 and 1979 and aggregated into six two-month periods, a peak in the incidence was found in May–June for males and September–October for females (Polednak, 1985). The patterns were generally dissimilar to those for melanomas of the skin, although the bi-monthly incidence of ocular melanoma diagnoses among males bore some resemblance to that for cutaneous melanoma of the trunk. Our findings, however, do not indicate such a similarity among males in the SEER data (Schwartz *et al.*, 1987).

Neither of the previous investigations analyzed the seasonal patterns by anatomic site within the eye. In this study melanomas coded as arising in the eyeball and those not coded to a specific site exhibited the strongest seasonal variation, but only among females did the patterns at all resemble those for cutaneous melanomas in the SEER data (Schwartz *et al.*, 1987).

It has been proposed that the peaks of cutaneous melanoma incidence in the summer months, which correspond to peaks in the intensity of ultraviolet radiation, represent a late-stage promotional effect of sunlight on *in-situ* or early invasive disease (Armstrong & English, 1988). To the degree that this is true, that similar variation has not been observed for ocular melanoma as a whole may suggest that sunlight does not play a similar role in this disease. However, for melanomas of the eye the absence of seasonal variation may represent, to some extent, an artefact of the natural history of the disease. Uveal melanomas most often come to diagnosis as a result of pain or loss of vision (Shields, 1983). If the incidence of these tumours truly increases during the summer, but clinical detection of some fraction of the cases is delayed (due to variation in tumour growth), a seasonal pattern similar to that observed for cutaneous melanomas might be obscured. It is also possible that detection of any seasonal patterns in ocular melanoma incidence requires the analysis of a larger number of tumours (although the present study involved substantially more cases than either of the previous investigations).

Whether or not melanomas arising in specific uveal structures exhibit seasonal variation similar to cutaneous melanoma could only be partially addressed by this study. It has been proposed that, based on the heterogeneity of exposure to ultraviolet radiation among uveal tissue, only melanomas of the iris (and not those of the choroid or ciliary body) would be expected to show any association with indices of solar radiation (Lerman, 1985). Our findings for choroidal melanomas would be consistent with a limited degree of exposure of this tissue to a likely carcinogenic component of sunlight, but our results for melanomas of the eyeball are difficult to interpret since the ICD-O classification did not permit us to distinguish between melanomas of the ciliary body and iris. The strikingly different seasonal incidence of eyeball melanomas for males and females, however, argues against the likelihood that, had such distinction been possible, consistent patterns between the sexes would be observed for either ciliary body or iris melanomas.

The authors thank John A.H. Lee and Stephen Van Den Eeden for their helpful comments.

This research was supported in part by Grant No. 1-R35-CA39779 from the National Cancer Institute.

### References

ARMSTRONG, B.K. & ENGLISH D.R. (1988). The epidemiology of acquired melanocytic naevi and their relationship to malignant melanoma. *Pigment Cell.*, **9**, 27.

GALLAGHER, R.P., ELWOOD, J.M., ROOTMAN, J. & 4 others (1985). Risk factors for ocular melanoma: Western Canada Melanoma Study. *J. Natl Cancer Inst.*, **74**, 775.

HEWITT, D., MILNER, J., CSIMA, A. & PAKULA, A. (1971). On Edwards' criterion of seasonality and a non-parametric alternative. *Br. J. Prev. Soc. Med.*, **25**, 174.

LERMAN, S. (1986). Sunlight and intraocular melanoma (letter). *N. Engl. J. Med.*, **314**, 712.

OSTERLIND, A. (1987). Trends in incidence of ocular malignant melanoma in Denmark 1943–1982. *Int. J. Cancer*, **40**, 161.

POLEDNAK, A.P. (1984). Seasonal patterns in the diagnosis of malignant melanoma of the skin and eye in Upstate New York. *Cancer*, **54**, 2587.

ROGER, J.H. (1977). A significance test for cyclic trends in incidence data. *Biometrics*, **64**, 152.

SCHWARTZ, S.M., ARMSTRONG, B.K. & WEISS, N.S. (1987). Seasonal variation in the incidence of cutaneous malignant melanoma: An analysis by body site and histologic type. *Am. J. Epidemiol.*, **126**, 104.

SCOTTO, J., FRAUMENI, J. & LEE, J.A.H. (1976). Melanoma of the eye and other noncutaneous sites: Epidemiologic aspects. *J. Natl Cancer Inst.*, **56**, 489.

SCOTTO, J. & NAM, J.-M. (1980). Skin melanoma and seasonal patterns. *Am. J. Epidemiol.*, **111**, 309.

SHIELDS, J.A. (1983). *Diagnosis and Management of Intraocular Tumors*. The C.V. Mosby Co.: St. Louis.

STRICKLAND, D. & LEE, J.A.H. (1981). Melanoma of eye: Stability of rates. *Am. J. Epidemiol.*, **113**, 700.

SWERDLOW, A.J. (1983). Epidemiology of melanoma of the eye in the Oxford region, 1952–1978. *Br. J. Cancer*, **47**, 311.

SWERDLOW, A.J. (1985). Seasonality of presentation of cutaneous melanoma, squamous cell cancer and basal cell cancer in the Oxford region. *Br. J. Cancer*, **52**, 893.

TUCKER, M.A., SHIELDS, J.A., HARTGE, P., AUGSBERGER, J. HOOVER, R.N. & FRAUMENI, J.F. (1985). Sunlight exposure as risk factor for intraocular melanoma. *N. Engl. J. Med.* **313**, 789.

WORLD HEALTH ORGANIZATION (1976). *International Classification of Diseases for Oncology*. Geneva: Switzerland.

YOUNG, J.L., PERCY, C. & ASIRE, A.J. (1981). Surveillance, epidemiology and end results: Incidence and mortality data, 1973–1977. *Natl Cancer Inst. Mongr.*, **57**, 1.